# Effectiveness of Conversational Agents (Virtual Assistants) in Health Care: Protocol for a Systematic Review

**DOI:** 10.2196/16934

**Published:** 2020-03-09

**Authors:** Caroline de Cock, Madison Milne-Ives, Michelle Helena van Velthoven, Abrar Alturkistani, Ching Lam, Edward Meinert

**Affiliations:** 1 Digitally Enabled Preventative Health Research Group Department of Paediatrics University of Oxford Oxford United Kingdom; 2 Department of Primary Care and Public Health Imperial College London London United Kingdom; 3 Institute of Biomedical Engineering Department of Engineering Science University of Oxford Oxford United Kingdom

**Keywords:** conversational agent, chatbot, voice recognition software, speech recognition software, artificial intelligence, virtual health care, avatar, virtual assistant, virtual nursing, virtual coach, intelligent assistant, digital health

## Abstract

**Background:**

Conversational agents (also known as chatbots) have evolved in recent decades to become multimodal, multifunctional platforms with potential to automate a diverse range of health-related activities supporting the general public, patients, and physicians. Multiple studies have reported the development of these agents, and recent systematic reviews have described the scope of use of conversational agents in health care. However, there is scarce research on the effectiveness of these systems; thus, their viability and applicability are unclear.

**Objective:**

The objective of this systematic review is to assess the effectiveness of conversational agents in health care and to identify limitations, adverse events, and areas for future investigation of these agents.

**Methods:**

The Preferred Reporting Items for Systematic Reviews and Meta-Analyses Protocols will be used to structure this protocol. The focus of the systematic review is guided by a population, intervention, comparator, and outcome framework. A systematic search of the PubMed (Medline), EMBASE, CINAHL, and Web of Science databases will be conducted. Two authors will independently screen the titles and abstracts of the identified references and select studies according to the eligibility criteria. Any discrepancies will then be discussed and resolved. Two reviewers will independently extract and validate data from the included studies into a standardized form and conduct quality appraisal.

**Results:**

As of January 2020, we have begun a preliminary literature search and piloting of the study selection process.

**Conclusions:**

This systematic review aims to clarify the effectiveness, limitations, and future applications of conversational agents in health care. Our findings may be useful to inform the future development of conversational agents and promote the personalization of patient care.

**International Registered Report Identifier (IRRID):**

PRR1-10.2196/16934

## Introduction

Digital technologies are driving transformation in the health sector and show promise in contributing to the resolution of major challenges facing health care systems worldwide, including the provision of personalized medicine, prevention of chronic conditions, care of an increasingly elderly population, and provision of health care to hard-to-reach populations. Intelligent digital platforms with a conversational user interface (ie, conversational agents) constitute a representative technology that has been investigated in these contexts [1-4]. Conversational agents mimic human interaction using natural language processing to analyze user inputs and respond appropriately using human language via auditory or textual methods [5].

The first technology of this kind emerged in 1966, constituting a text-based platform that mimicked a psychotherapist, “ELIZA”, using prerecorded answers selected based upon user input [6]. Over the past two decades, developments in natural language processing and deep learning have contributed to the development of more sophisticated artificial intelligence technologies, many of which employ conversational functions. Current agents are available via multiple digital platforms, including telephones, mobile phones, tablets, and computers, and in many virtual formats such as chatbots, embodied conversational agents, and three-dimensional avatars [2,7,8]. The input channels have similarly expanded in recent years; notably, conversational agents have evolved to integrate movement analysis and gesture or eye movement recognition, which may enhance the user-agent interaction by integrating multimodal signals as is the case in human-human interactions [9]. Within the health care field, conversational agents have been designed to automate specialized tasks to support health care professionals, patients, or at-risk populations [2,10-12]. The investigated uses for these systems include triage, diagnostics, counseling, health promotion, and training of health care professionals [1,4,11-16]. The widespread availability of the digital platforms through which these conversational agents operate enables populations with limited health provision or health literacy to access these services [14,17]. Finally, these agents are helping to provide patient-centered care by increasing the patients’ involvement in their health care and decision making [2,17,18]. Personalization features have also been integrated into conversational agents to improve user satisfaction, user engagement, and dialogue quality [19].

Despite a wealth of literature on conversational agents and their application to health care, the majority of reviews on the topic focus on a specific therapy area or function, whereas few reviews have comprehensively examined the overall scope and progress in the field [20-23]. Laranjo et al [24] conducted a systematic review of conversational agents in 2018, in which they investigated the characteristics, applications, and evaluation measures of conversational agents; however, this was limited to agents with unconstrained natural language input and systems that had been tested with human participants. Similarly, in 2019, Montenegro et al [25] surveyed the literature related to conversational agents applied to health care with a focus on their patterns, goals, and interactions. Although they described a general taxonomy detailing the functions and architecture, the implications for the users were not addressed.

There is a clear need to understand the effectiveness of current conversational agents to achieve their intended outcome and facilitate the user experience with these agents. This information can then be used to determine the direction that these technologies are most likely to follow in health care and identify the functions or populations that will derive the most benefit from these resources. Furthermore, these conversational agents have potential to alleviate current health care resource burdens by automating functions that previously required face-to-face interaction; thus, it is important to identify whether this is an observed outcome of the use of these technologies.

Thus, the aim of this systematic review is to evaluate the effectiveness and implications of conversational agents in health care. This review will focus on three main questions. First, are the intended health-related outcomes of current conversational agents being fulfilled, and does the effectiveness vary depending on the population or function of the agent? Second, what are the capabilities of health-focused conversational agents, and how might the availability of these agents impact the use of health care resources? Finally, what are the current limitations and gaps in the utility of conversational agents in the health care field that could inform future research?

## Methods

### Study Design

We will use the Population, Intervention, Comparator, Outcomes (PICO) template and the Preferred Reporting Items for Systematic Reviews and Meta-Analyses Protocols (PRISMA-P) guidelines [26] to identify appropriate Medical Subject Headings (MeSH) for the literature search and to structure the review. This systematic review will be composed of a literature search, article selection, data extraction, quality appraisal, data analysis, and data synthesis.

### Eligibility Criteria

The following PICO framework is based on our three main research questions stated above.

- Population: The population will include the general population, patients, students, and health care professionals of any age who have interacted with a conversational agent for any health-related purpose.
- Intervention: Interaction with a conversational agent that utilizes natural language processing via any interactive device.
- Comparator: No comparator is required for the studies to be included in this systematic review.
- Outcomes: The main health outcomes assessed will be those related to improvements in clinical, behavioral, and psychosocial parameters, along with health literacy, shared decision making, practical improvement in health care provision, or user-based evaluation outcomes, including acceptability, usability, engagement, and satisfaction.

### Search Strategy

We will search the following databases: PubMed (Medline), Embase, CINAHL, ACM Digital, and Web of Science. Key terms relating to conversational agents were extracted from an initial review of the literature, and specific search terms and strings were chosen in consultation with a medical librarian. Search terms will include MeSH terms and keywords related to conversational agents, natural language processing, health care, and evaluation. A draft of the search terms that will be used in this review are grouped into four themes in Table 1. All terms in the MeSH and keywords columns are included with the structure: (conversational agents [MeSH OR keywords] OR natural language processing [MeSH OR keywords]) AND (health [MeSH OR keywords] OR health-related education/training [MeSH OR keywords]) AND evaluation (MeSH OR keywords). We will adapt the search strategy as needed to return a breadth of papers without retrieving an unmanageably large number of irrelevant articles.

**Table 1:** Search terms.

Category	MeSH^a^	Keywords (title, abstract)
Conversational agent	Speech recognition software	“Conversational agent*” OR “embodied conversational agent” OR chatbot* OR avatar OR dialog* system OR “virtual assistan*” OR “virtual nurs*” OR virtual patient OR virtual coach* OR intelligent assistan* OR “relation* agent” OR “assistance technol*” OR “voice-based interfac*” OR “virtual coach” OR speech recognition software OR voice recognition software
Health	Healthcare facilities OR health services OR health communication OR health services accessibility OR delivery of healthcare OR health behavior OR simulation training OR health education OR health literacy OR patient acceptance of healthcare OR health knowledge, attitudes, practice OR asthma OR sex education OR exp aged OR exp counseling OR smoking cessation OR exp diet OR exp education, medical OR exp substance-related disorder OR social skills OR autism spectrum disorder OR patient education as topic OR exercise OR diabetes mellitus OR cardiovascular disease OR pulmonary disease, chronic obstructive	Health OR healthcare OR “health behavio?r” OR hospital OR exercis* OR diet OR healthcare delivery or healthcare access or simulation training or education or elderly care or sex* education or health literacy or counsel?ing or well-being or smoking cessation or cognitive dysfunction or mental health or social skills or autism spectrum disorder OR diabetes OR heart health OR chronic obstructive pulmonary disease OR COPD OR sun protection OR physical activity
Evaluation	Outcome assessment (Health Care) OR program evaluation OR feasibility studies OR pilot projects OR diffusion of innovation OR cost-benefit analysis OR reproducibility of results	Feasibil* OR usabil* OR evaluat* OR outcome* OR acceptability OR acceptance OR treatment adherence OR effectiv* OR adoption OR assess* OR user experience* OR efficacy OR utility OR utili?ation OR patient* acceptance OR patient* acceptability OR user* acceptance OR user* acceptability OR user* perce* patient* perce* or user perspective* OR patient* perspective* OR user* view* OR patient* view* OR cost*

^a^MeSH: Medical Subject Headings.

### Inclusion Criteria

The main criteria for inclusion will be interventional studies, including randomized controlled trials and non-randomized studies (eg, non-randomized controlled trials, before-and-after studies, and interrupted time-series studies), and observational studies, including cross-sectional surveys, cohort studies, and qualitative studies. Only studies published in English will be included.

There will be no restriction regarding the year of publication of studies to provide a comprehensive overview of the evolution of conversational agents in health care and the obstacles or successes that these agents have met to inform future research. Studies that evaluated at least one conversational agent will be included. Any population groups, geographical locations, or function intending to influence any aspect of physical or mental health or provide health-related education or training will be included to enable an assessment of the breadth of applications of conversational agents. Studies of conversational agents acquiring information via any input will be included; however, the agent must interact with a human user and adapt the response according to user input.

For an initial search, all study designs will be included; however, the studies included in the final review may be refined based on the initial results. An evaluation of the number of studies that are retrieved from an initial search may result in the exclusion of quasi-experimental trials or other study types.

### Exclusion Criteria

We will exclude studies that are not published in English and studies of conversational agent interventions that have no health-related function. Studies of conversational agents that utilize the Wizard of Oz technique, whereby a human operator is involved in response generation, or those not utilizing natural language processing will be excluded, as these do not constitute autonomous conversational agents. Conversational agents solely producing proactive communication will also be excluded (eg, reminder texts or electronic messages that cannot be responded to). Studies that report no evaluation of the conversational agent, such as papers discussing solely the design, development, or intention of the agent, will also be excluded.

### Screening and Article Selection

All articles identified from the database searches will be stored in the citation management software Mendeley (London, UK), which will be used to eliminate any duplicates. Two independent reviewers will screen the titles and abstracts of all studies. Studies that fail to meet the eligibility criteria will be excluded, with any disagreements being discussed until consensus is reached. The full text of the remaining articles will then be examined to determine final eligibility.

A PRISMA flow diagram will be used to record the details of the screening and selection process so that the study can be reproduced.

### Data Extraction

To extract data from the included studies, we will use a standardized Excel form that includes general information (title, author[s], year, country of study), study characteristics (study design, aim, study population, duration of study), risk of bias or quality assessment (depending on study design), details of the conversational agent (developer, architecture, intended application, design features), outcomes (including but not limited to health outcomes, user perception, usability, feasibility, and resource implications), limitations (including functional and user-reported limitations or potential improvements), and adverse events (such as data breaches, misinformation, or improper use). We will pilot the data extraction form on a small number of studies to develop the final data extraction form. One reviewer will review the full text of all the papers included in the final selection and extract data that will be validated by a second reviewer. Disagreements will be resolved by discussion, and if consensus cannot be reached, a third reviewer will be consulted.

### Quality Appraisal and Risk of Bias Assessment

After the final selection of the studies, two independent reviewers will assess the risk of bias of the included studies. If there is disagreement in judgment, the reviewers will discuss before consulting a third reviewer. The Cochrane Collaboration Risk of Bias tool will be used to assess any randomized controlled trials included in the review [27]. Since many of the included papers are anticipated to assess nonrandomized interventions, the Risk Of Bias in Non-randomized Studies of Interventions (ROBINS-I) will also be used [28]. The National Institutes of Health - National Heart, Lung, and Blood Institute’s quality assessment tool [29] will be used for observational cohort and cross-sectional studies. A table will be created summarizing the quality of all included studies.

### Data Analysis and Synthesis

It is unlikely that a meta-analysis will be feasible owing to the anticipated variety of study aims, methods, and reported outcomes. Therefore, we will conduct a descriptive analysis to summarize the extracted data. If possible, we will provide a narrative overview of results by subgroups. The discussion will synthesize the data to describe the effectiveness of current conversational agents as well as comment on the scope of the field; draw conclusions about their feasibility, usability, and acceptability; identify limitations and adverse events; and establish directions for future research and development.

## Results

As of January 2020, we have begun a preliminary literature search and piloting of the study selection process.

## Discussion

We will perform a systematic review and do not anticipate any issues with the implementation of the proposed protocol. This systematic review of the literature reporting the evaluation of conversational agents will offer new insight into the viability and progress of conversational agents in health care, and uncover challenges and limitations that have been encountered in order to inform the future development and evolution of these agents. This research will also add to the growing body of evidence and understanding of how health care can be further personalized. Our findings may also identify potential obstacles to the widespread implementation of these technologies, and aid in the future integration of conversational agents in clinical practice.

## Abbreviations

MeSH: Medical Subject Headings
PICO: Population, Intervention, Comparator, Outcomes
PRISMA-P: Preferred Reporting Items for Systematic Reviews and Meta-Analyses Protocols
ROBINS-I: Risk Of Bias in Non-randomized Studies of Interventions
